# Does Use of High-fidelity Simulation Improve Resident Physician Competency and Comfort Identifying and Managing Bradyarrhythmias?

**DOI:** 10.7759/cureus.6872

**Published:** 2020-02-04

**Authors:** Ghulam Rehman Mohyuddin, Amanda Jobe, Laura Thomas

**Affiliations:** 1 Internal Medicine, University of Kansas Medical Center, Kansas City, USA

**Keywords:** medical education, simulation, cardiology

## Abstract

Introduction: A workshop was designed to evaluate whether high-fidelity simulation with interactive case discussion could improve resident physician knowledge and comfort interpreting and managing bradyarrhythmias.

Methods: All the residents completed a pre-test and then participated in a one-hour interactive presentation, which included practice interpreting rhythm strips and 12-lead electrocardiograms. Forty-four residents were assigned to the intervention group and completed 10 simulated cases using a mannequin, a real defibrillator/external pacemaker, a medication cart, and a simulated telemetry monitor displaying real-time electrocardiograms under the guidance of two instructors. Seventeen residents were assigned to the control group and completed the same 10 cases using interactive discussion with the same instructors but without the use of the high-fidelity simulation models. All residents underwent post-testing.

Results: For the intervention group, the mean pre- and post-test knowledge scores were 13.93 and 17.28 (p=0.0001), and the mean pre- and post-test comfort scores were 2.92 and 4.24 (p=0.0001). For the control group, the mean pre- and post-test knowledge scores were 14.52 and 18.00 (p=0.005), and the mean pre- and post-test comfort scores were 2.97 and 4.35 (p=0.001). There were no statistically significant differences between pre-test and post-test knowledge and comfort scores for the two groups (p=0.633, p=0.421, p=0.177).

Conclusion: Interactive workshops help improve resident knowledge and comfort with identifying and managing bradycardias. The use of high-fidelity simulation models may not be superior to a similar interactive learning experience without the use of high-fidelity simulation tools.

## Introduction

Identification and management of bradyarrhythmias are the important skills for resident physicians to master because of the frequency with which these arrhythmias are encountered and the adverse consequences of misidentification and inappropriate management. In a study published in in 2009, Eslava and colleagues found low proficiency and low self-perceived confidence in electrocardiogram (ECG) interpretation among first-year internal medicine residents, highlighting a need for more robust ECG training. In their study, complete heart block was identified as a critical diagnosis. Residents achieved a mean score of only 0.23 out of 2 for this diagnosis [1].

Since there is evidence in the medical literature that demonstrates the need for more robust ECG training for medical residents, it is vital that a training curriculum be developed with the goal to enhance resident knowledge and promote retention of information. Over the past several years, there has been a transition towards active, case-based, and simulation-based learning in the world of medical education as evidence supports these learning styles may be superior to the traditional, lecture-based styles [2-4]. There have been studies published in recent years demonstrating case-based and simulation-based learning strategies as effective methods for training residents in the management of arrhythmias [5,6]. Our residency program has developed a case-based, simulation-based curriculum for the management of bradyarrhythmias.

The purpose of our study was to develop a structured workshop with interactive cases in an effort to improve resident competency and comfort in the management of bradyarrhythmias, and to determine whether the use of high-fidelity simulation mannequins leads to superior competency and comfort scores.

## Materials and methods

A total of 61 categorical (including medicine-psychiatry) internal medicine residents ranging from post-graduate year (PGY)-1 to PGY-5 participated in the bradyarrhythmia workshop. Approval from the institutional review board was obtained.

**Figure 1:**
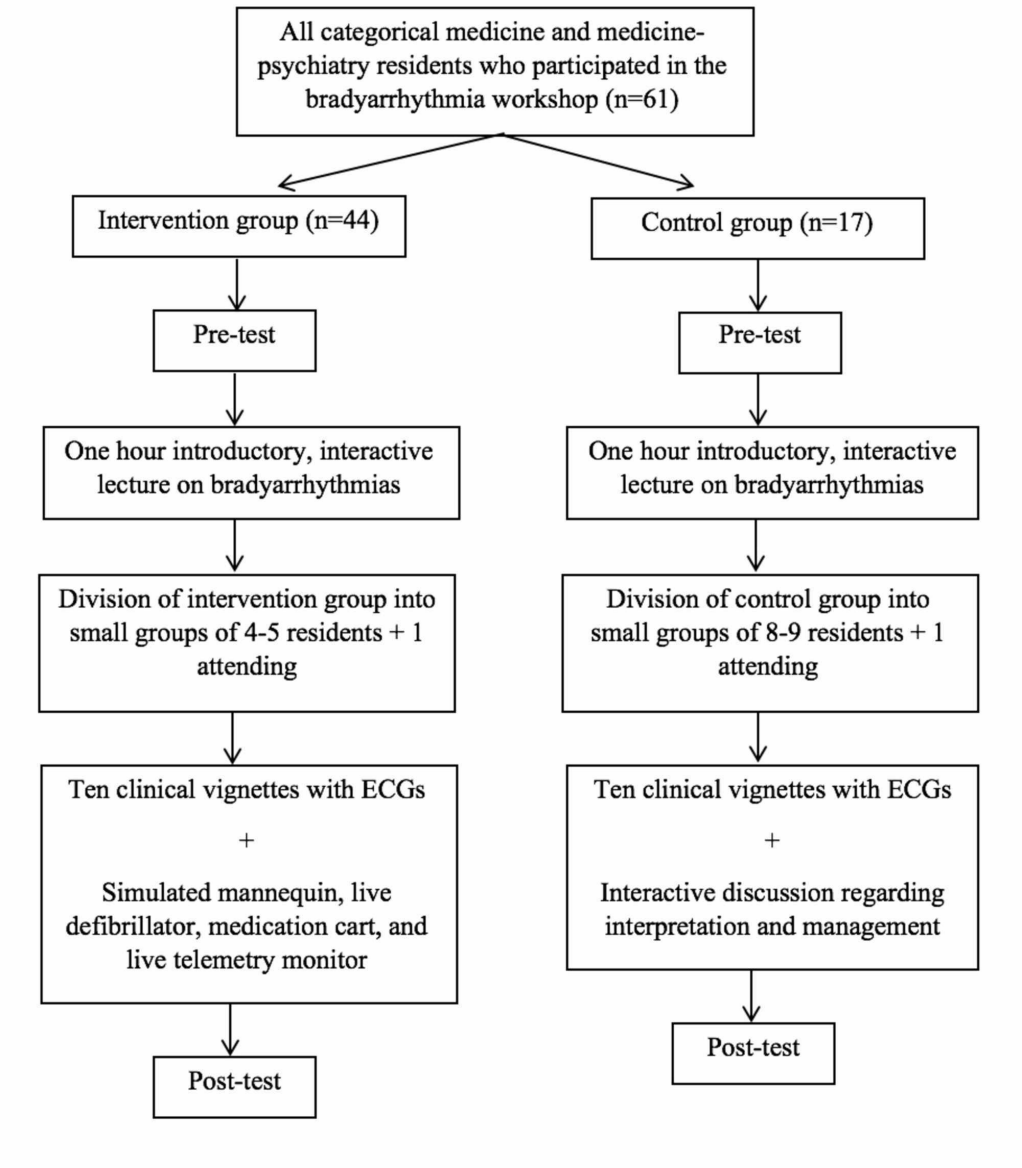
Consort diagram outlining study

The residents attended the two-hour workshop in eight separate groups. All residents completed a pre-test that assessed their competency and comfort in managing bradyarrhythmias. The competency portion of the pre-test consisted of five clinical vignettes with a corresponding ECG and associated questions in short answer format. The competency section was scored out of 20 possible points using a standardized scoring system and the grading was validated by a faculty cardiologist. Residents were also asked to gauge their comfort in managing bradyarrhythmias using a five-point Likert scale. After completion of the pre-test, all residents across both groups participated in a one-hour interactive session led by a faculty cardiologist who introduced the basics of common bradyarrhythmias and their management (Figure 1).

Forty-four residents were placed into the intervention group, which was further divided into small groups consisting of four to five residents and one attending physician. The intervention group was presented with 10 clinical vignettes and corresponding ECGs. Residents would have to identify the heart rhythm and proceed with management using a mannequin, live defibrillator with pacing capacity, medication cart, and live telemetry monitor that would change in real time based on resident intervention. 

Seventeen residents were placed into the control group, which was further divided into small groups consisting of eight to nine residents and one attending physician. The control group was presented with the same 10 clinical vignettes and corresponding ECGs. Residents would again have to identify the heart rhythm but would then participate in an interactive discussion regarding the appropriate management. There was no mannequin or live defibrillator for the control group, but case vignettes and an interactive discussion were ensured, similar to the intervention group. 

Following the workshop, all residents completed a post-test with competency questions identical to that of the pre-test and were again asked to gauge their comfort in the management of bradyarrhythmias using a five-point Likert scale. The post-test was scored in identical fashion to the pre-test. 

All statistical analysies were performed using SPSS version 25 (IBM Corp., Armonk, NY).

## Results

The goal of the study was to assess whether the interactive, case-based workshop improved resident competency and comfort in the identification and management of bradyarrhythmias, as well as to determine whether there was any difference in the competency and comfort scores between the intervention and control groups. The pre- and post-competency tests were scored out of a total 20 possible points, and the pre- and post-test comfort scores ranged from 1 to 5 based on a Likert scale. 

For the intervention group (n=44), the mean pre- and post-test competency scores were 13.93 and 17.28 (p=0.0001), and the mean pre- and post-test comfort level scores were 2.92 and 4.24 (p=0.0001). For the control group (n=17), the mean pre- and post-test competency scores were 14.52 and 18.00 (p=0.005), and the mean pre- and post-test comfort scores were 2.97 and 4.35 (p=0.001). Overall, for both the intervention and the control groups, resident competency and comfort scores improved from pre-test to post-test with statistical significance. 

In comparing the intervention group with the control group head to head, there was no statistically significant difference between the pre-test competency scores (p=0.633), the post-test competency scores (p=0.421), or comfort scores (p=0.177).

## Discussion

For both the intervention and the control groups, the competency and comfort scores improved from the pre-test to the post-test, which supports the use of active, case-based learning as an effective educational model for teaching interpretation and management of bradyarrhythmias. However, there was no difference in pre- or post-test competency scores and comfort scores when comparing the intervention group that utilized the high-fidelity simulation models with the control group head to head. 

While there is vast evidence in the medical literature to support simulation-based training, the use of high-fidelity simulation models may not always be superior [3,7]. We believe that a structured and interactive approach to learning is key, and our residents were able to improve their competency and comfort in managing bradyarrhythmias with or without the use of high-fidelity simulation models. 

Our bradyarrhythmia curriculum can easily be adopted across other academic institutions with or without the use of high-fidelity simulation models. These models come at a cost, are not available at all institutions, and may not always be practical or available depending on time and scheduling constraints. While the simulation models were more realistic, allowed residents to practice utilizing the pacing function on the defibrillator, and allowed residents to see telemetry changes in real time, they did not lead to superior outcomes on testing in our particular study. 

Our study has limitations in that our sample size was small and took place at a single institution. While we found no difference in competency and comfort scores between our intervention and control groups, we recognize this conclusion may not apply to other concepts, such as tachyarrhythmias, which involves different identification and management skills. Although there was no significant difference on test scores or comfort level, we also may have not adequately measured the best outcome. Ideally, the hope of simulation is to help transfer to real-life scenarios, which was not assessed in this study. The skills of using a pacer/defibrillator and adjusting treatment algorithm based on telemetry changes were unmeasured outcomes, which could possibly have been superior in the intervention group.

Future studies may also be valuable to re-assess competency and comfort scores one month after the workshop and compare retention rates between the intervention and the control groups. Also, connecting to real-life outcomes would also be beneficial, though this may be more difficult to obtain.

## Conclusions

Our experience shows that with implementation of a structured, interactive curriculum, residents can improve their competency and comfort in identifying and managing bradyarrhythmias with or without the use of high-fidelity simulation models. 
